# Negative affective stress reactivity: The dampening effect of snacking

**DOI:** 10.1002/smi.2788

**Published:** 2017-10-03

**Authors:** Saskia Wouters, Nele Jacobs, Mira Duif, Lilian Lechner, Viviane Thewissen

**Affiliations:** ^1^ Faculty of Psychology and Educational Sciences Open University of the Netherlands Heerlen The Netherlands; ^2^ Department of Psychiatry and Neuropsychology, European Graduate School for Neuroscience, SEARCH Maastricht University Medical Centre Maastricht The Netherlands

**Keywords:** daily hassles, ecological momentary assessment (EMA), experience sampling method (ESM), snacking, stress reactivity

## Abstract

The present study sets out to further elucidate the complex relationship between daily hassles, snacking, and negative affect (NA). The aim of the present study was to examine whether or not moment‐to‐moment energy intake from snacks moderates the association between momentary stress and NA. And, if so, can this moderating effect be replicated by using the amount of macronutrient intake (i.e., carbohydrates, fat, and protein) as moderator on the association between momentary stress and NA? Adults (N = 269), aged 20–50 years, participated in this study. Stress, NA, and snack intake were assessed 10 times a day for 7 consecutive days in daily life with an experience sampling smartphone application. Multilevel regression analyses were performed to assess the hypothesized associations. Our study revealed a dampening effect of snacking on negative affective stress reactivity. However, this dampening effect could not be replicated by the amount of macronutrient intake from snacks. On the contrary, the amount of carbohydrates has an enhancing effect on negative affective stress reactivity. In the end, our study suggests that the critical question is which mechanisms are decisive in the dampening role of snacking on stress reactivity. A multidisciplinary approach may provide a full perspective.

## 1 INTRODUCTION

Minor stressful daily events, such as being late for an appointment, missing a train, or having arguments with colleagues or family, are the annoying, worrying, frustrating, and stressful experiences that are embedded in our everyday life (Kanner, Coyne, Schaefer, & Lazarus, 1981). These minor stressful events or daily hassles are frequent and mostly inevitable and differ from major stressful life events (i.e., loss of job and death of spouse) that are less frequent and comprise a major change in individuals' circumstances or status. Stress research has traditionally been focused on major stressful life events (e.g., Dohrenwend & Dohrenwend, 1974; Warheit, 1979). This line of research is still applied (e.g., Flouri & Mavroveli, 2013; Phillips, Caroll, & Der, 2015; Yan, Li, & Sui, 2013). Studies examining major stressful life events and minor stressful daily events simultaneously, however, have demonstrated that the impact of frequent daily stressors on psychological distress should not be underestimated (e.g., Chamberlain & Zika, 1990; DeLongis, Folkman, & Lazarus, 1988; Heron, Bryan, Dougherty, & Chapman, 2013; Kanner et al., 1981; Larsson, Berglund, & Ohlsson, 2016; Monroe, 1983; Stefanek, Strohmeier, Fandrem, & Spiel, 2012). Even more, minor stressful daily events are known to have a cumulative negative impact on affect, behaviour, and (mental) health status of individuals (e.g., Falconier, Nussbeck, Bodenmann, Schneider, & Bradbury, 2015; Kanner et al., 1981; Larsson et al., 2016; Monroe, 1983; O'Connor, Jones, Conner, McMillan, & Ferguson, 2008).

Studies using the experience sampling method (ESM), also known as ecological momentary assessment (EMA), have repeatedly shown that minor stressful daily events are associated with an increase in negative affect (NA) and a general decrease in positive affect in clinical (e.g., Bylsma, Taylor‐Clift, & Rottenberg, 2011; Lardinois, Lataster, Mengelers, van Os, & Myin‐Germeys, 2011; Myin‐Germeys, Krabbendam, Delespaul, & van Os, 2003; Myin‐Germeys, van Os, Schwartz, Stone, & Delespaul, 2001; Peeters, Nicolson, Berkhof, Delespaul, & de Vries, 2003; Wichers et al., 2007) and nonclinical population samples (e.g., Jacobs et al., 2007; Marco, Neale, Schwartz, Shiffman, & Stone, 1999; van Eck, Nicolson, & Berkhof, 1998). In addition, it has been demonstrated that increased negative affective stress reactivity is a risk factor for the development of psychopathological disorders such as depression (Mezulis, Funasaki, Charbonneau, & Hyde, 2010; Morris, Ciesla, & Garber, 2010; Siegrist, 2008).

Daily life research has also demonstrated that increased consumption of hedonic “snack type” products, which are nutrient‐dense and high in sugar and fat, may be used in order to cope with the negative emotions associated with daily hassles (Newman, O'Connor, & Conner, 2007; O'Connor et al., 2008). Although studies in other settings confirm these findings (e.g., Cleobury & Tapper, 2014; Gibson, 2006; Groesz et al., 2012; Kandiah, Yake, Jones, & Meyer, 2006; O'Connor et al., 2008; Oliver & Wardle, 1999; Tomiyama, Dallman, & Epel, 2011; Torres & Nowson, 2007; Widaman, Witbracht, Forester, Laugero, & Keim*,* 2016; Zellner et al., 2006), not all individuals make use of this coping mechanism (e.g., Kandiah, Yake, & Willet, 2008; Macht, 2008). Research has pointed out that snacking may provide comfort or distraction from negative emotions associated with stress (e.g., Christensen, 1993; Gamble, Bava, & Wohlers, 2010; Macht, 2008; Macht & Simons, 2000; Spoor, Bekker, van Strien, & van Heck, 2007; Stice, Presnell, Shaw, & Rohde, 2005). Indeed, it has been demonstrated that particular types of consumptions may ameliorate stress via sensory or hedonic effects (Gibson, 2006). Studies within a 1‐day time span, have demonstrated that on a short‐term basis, the consumption of palatable food, high in fat and sugar, did decrease experimentally induced NA (Macht & Mueller, 2007; Wallis & Hetherington, 2009). However, 7‐day research using prospective food records and single daily mood measurements did not confirm these findings (Hendy, 2012). In addition, from a nutritional perspective, it has been demonstrated that macronutrients (particularly carbohydrates and fat) target the brain similar to opiates, providing a stress dampening effect (e.g., Cota, Tschöp, Horvath, & Levine, 2006; Groesz et al., 2012). Several biological mechanisms (e.g., serotonin hypothesis and endocrine hypothesis) have been postulated for the stress dampening effect of these specific macronutrients (e.g., Cota et al., 2006; Dallman et al., 2003; Wurtman & Wurtman, 1989). However, these findings have not always been confirmed (Benton, 2002).

The question arises whether snacking as a mechanism to cope with or distract from daily life stressors, could actually moderate (i.e., dampen) the association between stress and NA. And if so, can this moderating effect be replicated by using the amount of macronutrient intake (i.e., carbohydrates, fat, and protein) as moderator on the association between momentary stress and NA? Therefore, the present study sets out to extend our knowledge of the impact of snacking and its nutritional components on NA in response to daily hassles. As high‐energy snack intake may contribute to overweight and obesity, which are prominent risk factors for health problems (e.g., Guh et al., 2009; Torre et al., 2015), more knowledge may help to better tailor interventions addressing unhealthy snacking behaviour and increase their effectiveness.

To capture the variety of daily hassles, affective states, and eating occasions throughout the day, a momentary ecological assessment approach was used. Because in the individuals' natural environment all types of snacks are available in order to cope with minor stressful daily events, the present study includes all types of between‐meal snacks including beverages and healthy snacks such as fruit and vegetables. Repetitive assessments of minor stressful daily events, NA, and snack intake allow to investigate the moderating effect of snacking (whether or not energy intake was consumed) on negative affective stress reactivity. Additionally, as the main building blocks of energy intake from snacks are carbohydrates, fat, and protein, the moderating effects of these macronutrients on negative affective stress reactivity were also examined.

In sum, the current study aims to examine whether or not momentary energy intake from snacks and its amount of macronutrients (i.e., carbohydrates [grams], fat [grams], and protein [grams]) moderate the association between minor stressful daily events and NA. It is hypothesized that (a) energy intake from snacks dampens the negative affective reactivity to daily hassles. In addition, it is expected that this effect is replicated by its macronutrients: (b) the amount of momentary carbohydrate intake and (c) momentary fat intake. It is hypothesized that this dampening effect cannot be replicated by (d) the amount of momentary protein intake.

## 2 MATERIAL AND METHODS

### 2.1 Sample

Participants were recruited throughout the Netherlands via social media, websites, newsletters, and within the networks of several master thesis students at the Open University of the Netherlands. Master students at this university are adults with heterogeneity in variables such as previous education, age, marital status, employment status, and income.

Individuals had to be 20–50 years of age to be included in the analyses, as research has shown the largest increase in overweight individuals in recent years within this age group in the Netherlands (CBS Statline, 2014; Nationaal Kompas Volksgezondheid, 2014). In addition, participants had to be in possession of an Android smartphone as the Snackimpuls app was designed for this platform. Exclusion criteria were currently following a diet, being treated for eating disorders in the present or the past, participating outside of the research period (see procedure), and unfamiliarity with the Dutch language.

All participants agreed to an informed consent. This study was approved by the Ethics Committee of the Open University of the Netherlands.

### 2.2 Procedure

The research took place in the Netherlands in the period from mid‐October 2012 until early December 2013. Respondents were enrolled for 1 week during the research period, were instructed to take part during a regular week excluding holidays, and to maintain their usual food intake.

The Snackimpuls app and the Snackimpuls website, which contain participant's information and instructions, were created for this study by the Open University of the Netherlands. Recruited participants were referred to the website to consult information about the study, including instructions for downloading and installing the Snackimpuls smartphone app. After registration at the website, participants automatically received an email with a link to an online questionnaire. Having completed this questionnaire, participants automatically received login credentials for the free smartphone app. A demo version was included in the smartphone app as a training opportunity on the day prior to the assessment period.

To collect multiple assessments of stress, current emotions, self‐esteem, situational and social context, and between‐meal snack intake, respondents repetitively (10 times a day on seven consecutive days) answered a short questionnaire (37 items) on their smartphone. This questionnaire took approximately 5 min to complete. In addition, participants daily answered a brief self‐initiated questionnaire on their smartphone after waking up (four items) and before going to bed (10 items). After waking up, respondents' quality of sleep was assessed. Before going to bed, questions were asked about respondents' reflective assessments of the past day, and between‐meal snack intake was assessed one last time to cover late night snacking. Finally, participants were instructed to synchronize the data on their smartphone with the main server of the Snackimpuls project at the end of their research period. To enhance compliance, participants were able to contact a member of the research team by email in case of questions or problems. In addition, three Android tablets were raffled off amongst the participants as a reward, and participants received personal feedback based on their individual scores regarding eating behaviour (Dutch Eating Behavior Questionnaire, van Strien, Frijters, Bergers, & Defares, 1986), daily activities, and affective states (Snackimpuls app).

### 2.3 Instruments

Two instruments were used to collect the data. First, at baseline, an online composite questionnaire was used to collect data on demographics. Subsequently, a smartphone application was used to collect repetitive data of event‐related stress, NA, and between‐meal snack intake.

Because this study was part of a larger study to investigate determinants of between‐meal snacking in daily life (Wouters, Jacobs, Duif, Lechner, & Thewissen, in press; Wouters, Thewissen, Duif, Lechner, & Jacobs, 2016; Wouters, Thewissen, Duif, Lechner, & Jacobs, under review; Wouters, Thewissen, Zamani, Lechner, & Jacobs, 2013), other concepts (not used in the current study) were assessed as well with the online questionnaire (e.g., eating style, habit, and personality) and with the Snackimpuls app (e.g., ego depletion and quality of sleep).

### 2.4 Online composite questionnaire

#### 2.4.1 Demographics

Demographic variables such as age, weight, height, gender, marital status, and level of education were assessed. In this study, level of education was categorized as high education (higher vocational or academic education) and low to middle education (none, elementary school or lower general education, intermediate general education, intermediate vocational education, higher general secondary education, or pre‐university education). Body mass index (BMI) was calculated as weight (kg) divided by height (m) squared.

### 2.5 The experience sampling smartphone application

Stress, NA, and between‐meal snack intake were assessed in daily life with the ESM (Csikszentmihalyi & Larson, 1987; Hektner, Schmidt, & Csikszentmihalyi, 2007), a validated structured self‐assessment diary method. General guidelines have emerged with regard to the number of daily signals in ESM research. The most common range is 4–10 prompts a day (Conner & Lehman, 2012). Delespaul (1992) recommends a maximum of six signals a day when the sampling period is longer than 3 weeks. Within these boundaries, the Snackimpuls app produced 10 quasi‐random (with an average interval of 90 min) audio signals (beeps) a day for seven consecutive days between 7:30 a.m. and 10:30 p.m. Respondents were instructed to complete the reports immediately after the signal.

Consistent with previous ESM studies (Bylsma et al., 2011; Jacobs et al., 2007; Lardinois et al., 2011; Myin‐Germeys, Krabbendam, & Delespaul, 2003; Myin‐Germeys et al., 2001; Wichers et al., 2007), stress was defined as the subjective appraisals of stressfulness of minor daily events (i.e., event‐related stress). Participants were instructed to think about the most important event since the previous beep or, if no previous beep was emitted yet, since waking up. Subsequently, the participants' appreciation of the event was rated on a 7‐point Likert scale, ranging from “very unpleasant” through “neutral” to “very pleasant.” Afterwards, this item was recoded in order to facilitate interpretation: the higher the score, the higher the subjective appraisal of stress.

NA was assessed with the items “I feel insecure,” “I feel anxious,” “I feel down,” “I feel nervous,” “I feel bored,” and “I feel rushed” that were derived from the Positive Affect and Negative Affect Schedule ([PANAS] Watson, Clark, & Tellegen, 1988) and previous ESM studies (Geschwind et al., 2010; Jacobs et al., 2007; Peeters et al., 2003; Thewissen et al., 2011; Wichers et al., 2012). The items were rated on 7‐point Likert scales, ranging from 1 (*not at all*) to 7 (*very*). Momentary NA was defined as the mean score of the NA items for each individual per beep. The higher the score, the higher the momentary NA. The reliability estimate (Cronbach's α) was calculated at person level. In the current study, the NA scale had a very high internal consistency (α = .92).

With regard to between‐meal snacking, participants answered the question: “Did you eat or drink anything between meals since the last beep?” by replying “Yes” or “No”. If the reply was negative, this was equated with 0 kcal. If the answer was affirmative, they were asked to report every product consumed and its quantity. To help participants facilitate the recording of snack intake, the Snackimpuls app had a built‐in search function. This search function consulted a food composition table based on the scientifically accepted Dutch Food Composition Database (Rijksinstituut Volksgezondheid en Milieu [RIVM], 2011). For every reported snack, participants chose between two quantity options. Natural products, such as an apple, and products with standardized quantities, such as a Mars candy bar, could be reported either per piece or in grams (for solid foods) or millilitres (for fluids). Products with undetermined quantities such as yoghurt or tea could be reported in relevant household measures (i.e., a bowl or a cup) or in grams or millilitres. In addition to assessments prompted by the audio signals, between‐meal snack intake was also assessed by a daily self‐initiated short questionnaire just before going to bed, using the same reporting procedure as described above. The snack intake was automatically converted into total momentary energy intake (kcals) and its macronutrients carbohydrates (g), fat (g), and protein (g). This information was not visible to the participants. Products which were not available in the search facility could easily be added by the participants using the keyboard of their smartphone. These self‐added reported snacks were converted into their corresponding energy intake (kcals) and its macronutrients (g) by two independent researchers. The kilocalories and the macronutrients (g) for these products were extracted from the scientifically accepted Dutch Food Composition Database (Rijksinstituut Volksgezondheid en Milieu, 2011). If reported products were not available in the Dutch Food Composition Database, the database of Netherlands Nutrition Centre (2013) was consulted. Inter‐rater reliability yielded high correlation coefficients on kilocalories (*r* = .95, *p <* .01), carbohydrates (*r* = .95, *p <* .01), fat (*r* = .98, *p <* .01), and protein (*r* = .95, *p <* .01). A pilot study has demonstrated the feasibility and usability of the Snackimpuls app (Wouters et al., 2013).

### 2.6 Statistical analyses

In the analyses, NA on the current beep was the dependent variable, and the subjective appraised stressfulness of the most salient minor event since the previous beep was the independent variable. Negative affective stress reactivity was conceptualized as the association between stress and NA.

Snack intake was also measured retrospectively and was converted into its corresponding energy intake (kcals) and its macronutrients (g). Energy intake and macronutrients of all reported items since the previous beep were calculated into one score for one time point.

Because ESM data have a hierarchical structure with repeated momentary measurements (Level 1) for each participant (Level 2), multilevel linear techniques were used. Statistical analyses were performed to evaluate which model best fitted the data (i.e., fixed or random slopes). Subsequently, multilevel linear regression analyses were carried out using the xtmixed procedure in STATA/MP Version 11 (StataCorp, 2009). The key variables were standardized prior to the analyses. After standardization, the associations could be assessed directly, and their importance was evaluated by using the calculated regression coefficients (β). All analyses were adjusted for the potential confounders: gender, age, level of education, and BMI. The level of significance was defined at *p* < .05 for the main analyses and *p* < .10 for the interaction terms.

First, a multilevel regression analysis was performed to replicate earlier findings with regard to the positive association between the appraised stressfulness of minor daily events and NA (e.g., Jacobs et al., 2007; Jacobs et al., 2006; Marco et al., 1999; van Eck et al., 1998). To test the hypothesis whether or not energy intake from snacks dampens negative affective stress reactivity, a dichotomous variable, indicating whether or not energy intake was consumed (snacking: yes or no), was created. In addition, an interaction variable (Stress × Snacking) was included in the multilevel regression analysis assessing the moderating role of snacking. Additional analyses, stratified by snack intake respectively no snack intake, were performed.

To determine at beep level whether negative affective stress reactivity was moderated by the amount of carbohydrate (g), fat (g), or protein intake (g) from snacks, interaction variables (Stress × Carbohydrates; Stress × Fat; and Stress × Protein) were created. Separate multilevel regression analyses were performed to assess the moderating role of the macronutrients. The analyses with regard to each of the macronutrients were adjusted for the other two macronutrients. For significant interaction terms, additional multilevel regression analyses, stratified by tertiles of the macronutrient intake, were performed.

Dropout analyses were conducted (two‐sample Wilcoxon rank‐sum [Mann–Whitney] tests) to investigate significant differences in age and BMI between participants who finished the study and the dropouts. Effect sizes were expressed as correlation coefficients (Pearson's *r*; Field, 2005). In addition, chi‐square analyses were conducted to investigate significant differences in the distribution of gender and level of education between these two groups.

## 3 RESULTS

**Figure 1:**
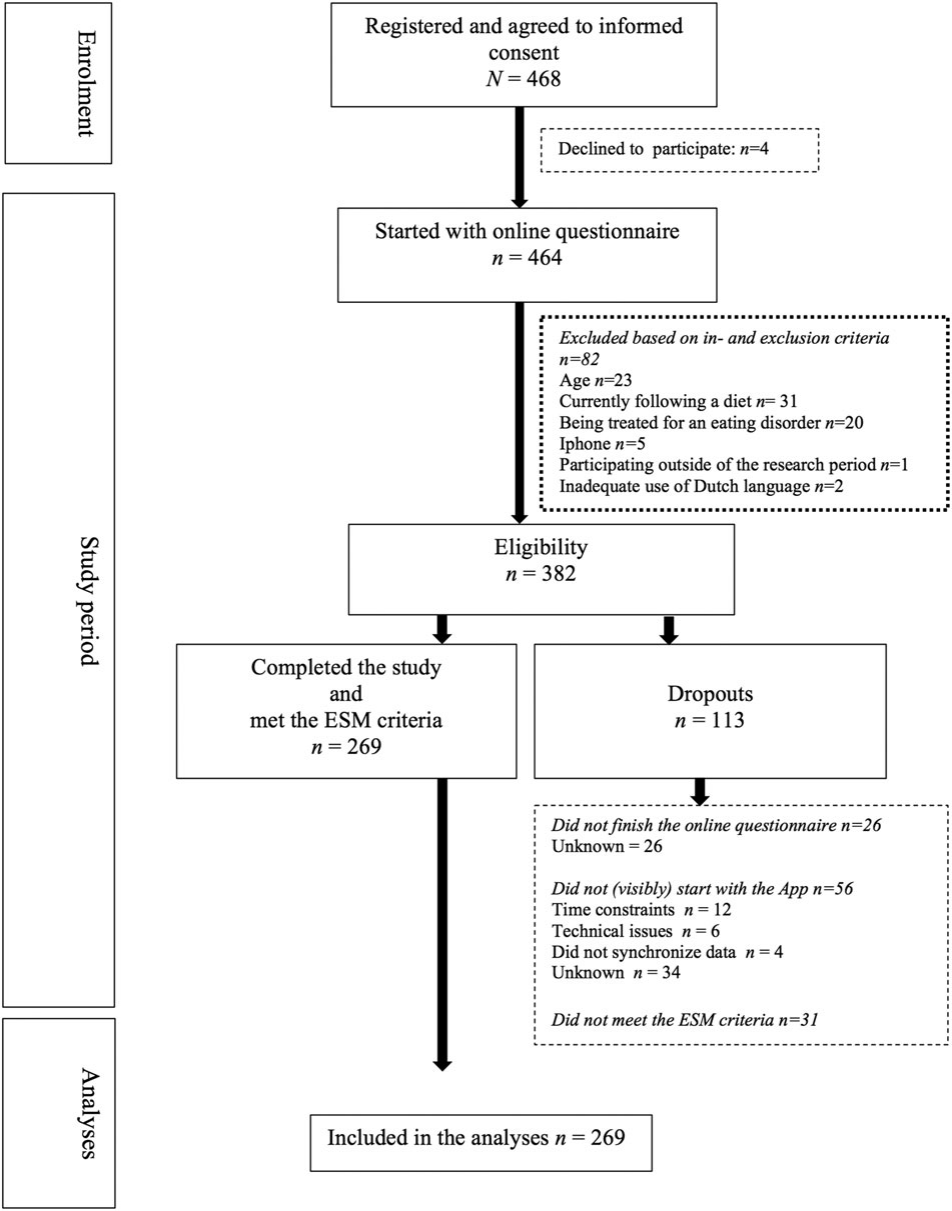
Study flowchart. ESM = experience sampling method

Of the total sample that participated in the study (*N* = 464), 82 respondents (18%) did not meet the inclusion criteria. Of the eligible sample (*n* = 382), 113 participants (30%) dropped out and 269 participants (70%) completed the study (Figure 1). Missings in this study occurred at beep level, which is a known phenomenon in ESM research (Silvia, Kwapil, Eddington, & Brown, 2013). Participants were instructed to complete their reports immediately after the beep, to minimize memory distortion. Reports not completed within 15 min after the beep were considered invalid. Data from the eligible participants were considered valid if they had reported at least 33% of the total number of assessments with the app during the 7‐day research period (Delespaul, 1995). Participants who did not meet this criterium were considered to be dropouts. Data from all the completers were included in the analyses.

Dropouts did not differ from the participants who finished the study (*n* = 269) with regard to BMI (*Z* = −.26, *p* = .80). Moreover, no significant differences were found in the distribution of gender, χ^2^(1, *n* = 382) = .91, *p* = .34, and level of education, χ^2^(1, *n* = 382) = .01, *p* = .94, between both groups. However, dropouts were slightly younger (mean age 33 vs. 35 years; *Z* = 2.67, *p* = .01). The effect size of this finding was small (*r* = .14). Mean age of the completers (197 females [73%] and 72 males [27%]) was 35 years (*SD* = 8.91), and mean BMI was 24 (*SD* = 4.00). Of the participants, 61% had a higher vocational or academic degree (Table 1). Aggregated over participants' means, mean NA was 1.55 (*SD* = 0.58, range 1–4.27). Mean subjective appraisal of stress was 3.54 (*SD* = 0.52, range 1.12–5.14).

**Table 1:** Individual characteristics and mean momentary negative affect and event‐related (un)pleasantness scores (*n* = 269)

Characteristic	*n*	%	*M* (*SD*)	Range	
Gender	269				
Male	72	27			
Female	197	73			
Age	269		35.42 (8.91)	20–50	
BMI	267		24.39 (4.00)	17–43	
<18.5	4	1			
18.5 ≤ BMI < 25	162	61			
25 ≤ BMI < 30	79	30			
≥30	22	8			
Education	269				
High	163	61			
Low to middle	106	39			

Variable	*n*	%	*M* (*SD*)	Range	Cronbach's α
Mean momentary NA	269		1.55 (0.58)	1–4.27	.92
Mean event‐related stress	269		3.54 (0.52)	1.12–5.14	

*Note*. *M* = mean; *SD* = standard deviation; BMI = body mass index; NA = negative affect. High education = higher vocational or academic education. Low to middle education = none, elementary school, lower general education, intermediate general and intermediate vocational education, higher general secondary or pre‐university education.

Snack intake could be reported 11 times a day (10 momentary reports and the final report just before going to bed to cover late night snacking). Study participants yielded 14,330 momentary reports, 69% of the maximum number of assessments (11 reports × 7 days × 269 participants) with the Snackimpuls app. In 7,174 assessments (50%), participants indicated that they did consume between‐meal snacks. However, information on snack intake was missing at 572 (8%) of the 7,174 assessments: Although respondents indicated they did consume something between‐meals, no products were reported (Table 2). In these instances, the missing snack reports and the corresponding measures (i.e., carbohydrates, fat, and protein) were treated as missings.

**Table 2:** Momentary reports (*N* = 14,330)

	*N*
Snack consumption: no	7,156
Snack consumption: yes	7,174
With kilocalories	5,198
Without kilocalories (e.g., water and black coffee)	1,404
No products reported	572

The 6,602 momentary reports of between‐meal snack intake (with and without kilocalories; Table 2) comprised 11,520 reported between‐meal snacks of which 9,593 snacks (83%) were reported with the search facility of the app (e.g., apple pie, cake, biscuit, and coffee with milk and sugar) and 1,927 snacks (17%) were reported manually (e.g., cheesecake and wiener mélange). Manually reported snacks also consisted of products that were present in the search function but which were not recognized due to spelling differences.

If snacks were reported, between‐meal snacking resulted in a mean energy intake between two beeps of 162 kcal (*SD* = 216) per respondent, mainly originating from a mean momentary carbohydrate intake of 21 g (*SD* = 26), a mean momentary fat intake of 5 g (*SD* = 11), and a mean momentary protein intake of 3 g (*SD* = 7).

Statistical analysis showed that the model with random slopes better fitted the data according to the Akaike information criterion (Hox, 2010). Therefore, a multilevel regression model, assuming unequal associations for each individual (random slopes) and random intercepts, was applied.

#### Negative affective stress reactivity

A preliminary analysis showed a significant positive association between momentary subjective appraisal of stress and NA (β [*SE*] = .15 [.01], *p* < .01). The higher the momentary subjective appraisal of stress, the higher the momentary NA.

#### The moderation of snacking

Results of the interaction analysis revealed a significant interaction between momentary subjective appraisal of stress and snacking (β [*SE*] = −.05 [.01], *p* = <.01) in association with NA. Additional multilevel regression analyses, stratified by snack intake respectively no snack intake, showed a slight dampening effect of snack intake on the association between stress and NA (β [*SE*] = .14 [.02], *p* < .01) compared to no snack intake (β [*SE*] = .17 [.02], *p* < .01).

#### The moderation of macronutrients

Results of the interaction analyses revealed a significant interaction between momentary subjective appraisal of stress and the amount of carbohydrate intake (β [*SE*] = .04 [.02], *p* = .048) in association with NA. To enable additional analyses, carbohydrate intake was divided into tertiles (.01–13.44 g; 13.44–29.71 g; and 29.71 to and including 455 g). Multilevel regression analyses, stratified by tertiles of carbohydrate intake, showed an enhancing effect of carbohydrate intake on the association between stress and NA in Tertile 1 (β [*SE*] = .10 [.02], *p* < .01), Tertile 2 (β [*SE*] = .12 [.02], *p* < .01), and Tertile 3 (β [*SE*] = .18 [.02], *p* < .01). The higher the tertile of carbohydrate intake, the higher the momentary NA in response to the momentary subjective appraisal of stress. No significant interaction was found between momentary subjective appraisal of stress and the amount of fat intake (β [*SE*] = .00 [.02], *p* = .81), respectively protein intake (β [*SE*] = .01 [.02], *p* = .62) in association with NA.

## 4 DISCUSSION

The aim of the present study was to examine whether or not moment‐to‐moment energy intake from snacks moderates the association between momentary stress and NA. And, if so, can this moderating effect be replicated by using the amount of macronutrient intake (i.e., carbohydrates, fat, and protein) as moderator on the association between momentary stress and NA?

Results of the preliminary analysis replicated the significant positive association between momentary stress and momentary NA: the higher the subjective stress appraisal, the higher NA. This confirms previous findings of ESM studies in similar samples (e.g., Jacobs et al., 2007; Marco et al., 1999; van Eck et al., 1998). In addition, results of the interaction analysis using snack intake (yes or no) as a moderator, revealed a significant interaction between momentary stress and snacking in association with NA. Additional multilevel regression analyses, stratified by snack intake respectively no snack intake, showed a slight dampening effect of snack intake on negative affective stress reactivity compared to no snack intake. Although the effect size of this finding was small, this may still be relevant given the interaction with very frequently occurring minor stressful events in daily life. If individuals are reducing their repetitive daily stress response (i.e., an increase in NA) by energy intake, this may have major cumulative health‐related effects due to their high frequency.

Moreover, contrarily to our expectations, results of the interaction analyses using the macronutrients as moderators, revealed a significant interaction between momentary stress and carbohydrate intake in association with NA. The higher the tertile of carbohydrate intake since the previous beep, the higher the momentary NA in response to momentary stress. These findings seem to add to previous research demonstrating that, in contrast to consuming healthy snacks, unhealthy, sugar‐rich, snack intake promotes negative affective states (Singh, 2014). It has been pointed out that this may strengthen the condition for increased stress sensitivity that may lead to repetitive unhealthy snacking (Singh, 2014). In addition, experimental research in female samples has demonstrated that although unhealthy snack intake may evoke positive emotions at the time of consumption, this may shortly thereafter be replaced by feelings of guilt due to a negative self‐evaluation related to giving into the temptation of unhealthy snacking (Macht & Dettmer, 2006; Macht, Gerer, & Ellgring, 2003; Steenhuis, 2009). Moreover, from a nutritional perspective, our findings seem to confirm previous research that opposes a mood dampening effect of serotonin. For instance, Benton (2002) has demonstrated that even small proportions of protein intake may annul the serotonergic stress dampening effects of carbohydrates. The latter seems in line with our broad definition of snacking that allowed for consumption of many different products that may diverge in composition. Finally, no significant interactions were found between momentary stress and the amount of fat—respectively protein intake in association with NA.

In sum, our study demonstrates that the consumption of snacks has a slight dampening effect on negative affective stress reactivity. Although this finding seems consistent with previous studies demonstrating that on a short‐term basis the consumption of highly palatable snacks did decrease experimentally induced NA (Macht & Mueller, 2007; Wallis & Hetherington, 2009), our study also shows that this dampening effect of snacking on stress reactivity cannot be replicated by its macronutritional components. On the contrary, the only macronutritional effect found was an increase of stress reactivity based on the amount of carbohydrates consumed. From a macronutritional perspective, this seems to indicate that consuming snacks high in carbohydrates is counterproductive for decreasing negative affective stress reactivity. Our study suggests that snacking, irrespective of the type of snack consumed, slightly dampens negative affective stress reactivity. Given the dampening role of snacking on negative affective stress reactivity, the critical question is which potential mechanisms are decisive in this dampening effect. An integrated multidisciplinary research approach involving neurochemical, nutritional, and psychological influences may provide a full perspective on the impact of nutrition on the association between stress and NA. Indeed, it may well be that sweet snacks provide more satisfaction or anticipated distraction (Desmet & Schifferstein, 2008; Gamble et al., 2010) from daily life stressors and thereby, from a psychological perspective, contribute to the dampening effect from stress reactivity, whereas from a macronutritional perspective, our study shows that this effect is reversed. It could also be considered to perform some more complex analyses (i.e., mediated moderation or moderated mediation) because the association between stress and NA may also be mediated by snacking (e.g., Finch & Tomiyama, 2014; Köster & Mojet, 2015). More knowledge may help to better tailor interventions addressing unhealthy snacking behaviour and thereby increase their effectiveness.

Some limitations of this study have to be noted. A first concern is the issue of causality that relates to the moment at which between‐meal snacks were consumed. It remains unclear whether momentary between‐meal snacks were consumed before, during, or after the event took place. Therefore, it cannot be established whether the subjective appraisals of stress increased snack intake, or whether snack intake influenced the subjective appraisals of stress. In accordance, it remains unclear whether snack intake alleviates the impact of a stressful event on NA reactivity (when the snack is eaten after the event), or whether snack intake sensitizes a person for the effect of a NA reaction in response to a stressful event (when a snack is eaten before the event). However, the interpretation of stress, at least in part contributing to changes in food choice, has face validity (e.g., Greeno & Wing, 1994; Macht, 2008). Future research may consider examining the impact of snacking and its macronutrients on negative affective stress reactivity in a prospective framework to obviate this lack of clarity. A second concern is the broad definition of between‐meal snacks. Research has demonstrated that different types of consumptions may have differential effects on NA (Singh, 2014). Indeed, it has been demonstrated that for instance, chocolate may dampen tension (Cartwright et al., 2007; Fletcher, Pine, Woodbridge, & Nash, 2007; Macht & Mueller, 2007; Osman & Sobal, 2006; Parker, Parker, & Brotchie, 2006), whereas coffee, may induce anxiety (Acquas, Tanda, & Di Chiara, 2002; Rossi et al., 2010). However, because in daily life all types of consumptions may be consumed, our results lead us to the conclusion that we were still able to provide relevant findings on the impact of snacking on negative affective stress reactivity. Nevertheless, the differential effect of different types of consumptions remains an important endeavour for future research. A third concern is the exclusion of main meals in this study, which implies that the results of our analyses may not comprise all energy intake consumed. In addition, it is conceivable that healthy snacks could have another effect on the association between minor stressful events and NA than unhealthy snacks. However, an additional analysis on a subset of the data (*n* = 14,085) excluding beeps (*n* = 245), which only comprised healthy products (i.e., fruit and/or vegetables), showed similar findings. Moreover, as self‐reports were being used, we cannot be fully sure that participants followed the instructions to only report products consumed outside of the regular meals. Nevertheless, mean momentary energy intake between beeps and the reported products seem to indicate that individuals complied with the instructions.

Despite these limitations, several strengths of this study also have to be mentioned. First, ESM was used in assessing subjective appraisals of stressfulness of minor daily events, snack intake, and NA. Daily life research protocols such as ESM have demonstrated to be a valid and reliable method for capturing minor stressful events, behaviour, and affect in daily life (e.g., Csikszentmihalyi & Larson, 1987; van Eck et al., 1998). In addition, we conducted a comparison study (Wouters et al., 2016) to validate the measure of momentary kilocalories used in the current study. The comparison study showed that momentary energy intake from snacks as reported with the ESM smartphone app was comparable to the reports with a paper and pencil estimated diet diary. Second, compared to laboratory studies, ESM enhances the ecological validity of the findings (Hektner et al., 2007). Third, this is the first ESM study to address the moderating role of snacking (yes or no energy intake consumed) and the amount of macronutrients (i.e., carbohydrates, fat, and protein) on negative affective stress reactivity.

## 5 CONCLUSION

The findings of the present study provide support for the dampening role of snacking on negative affective stress reactivity. However, this effect cannot be replicated by its macronutritional components. In the end, the critical question is which mechanisms are decisive in this dampening effect. A multidisciplinary approach involving neurochemical, nutritional, and psychological influences may provide a full perspective of the dampening role of snacking on stress reactivity.
